# Evolving Indications of Transcatheter Aortic Valve Replacement—Where Are We Now, and Where Are We Going

**DOI:** 10.3390/jcm11113090

**Published:** 2022-05-30

**Authors:** Jules Mesnier, Vassili Panagides, Jorge Nuche, Josep Rodés-Cabau

**Affiliations:** Quebec Heart and Lung Institute, Laval University, Quebec City, QC G1V 4G5, Canada; mesnier.jules@gmail.com (J.M.); vassili.panagides@gmail.com (V.P.); jorge-nuche@hotmail.com (J.N.)

**Keywords:** transcatheter aortic valve replacement, aortic stenosis, aortic regurgitation, bicuspid valve, moderate aortic stenosis

## Abstract

Indications for transcatheter aortic valve replacement (TAVR) have steadily increased over the last decade since the first trials including inoperable or very high risk patients. Thus, TAVR is now the most common treatment of aortic valve stenosis in elderly patients (vs. surgical aortic valve replacement -SAVR-). In this review, we summarize the current indications of TAVR and explore future directions in which TAVR indications can expand.

## 1. Introduction

Transcatheter aortic valve replacement (TAVR) was initially restricted to patients with severe symptomatic aortic stenosis (AS) deemed at prohibitive risk for surgical aortic valve replacement (SAVR) [1]. One randomized trial at a time, TAVR showed its efficacy in high, intermediate and, recently, low surgical-risk patients for the management of severe degenerative AS [2,3,4,5,6,7]. As a result, almost twice as much TAVR procedures were performed compared to SAVR in the United States (US) in 2019 [8]. This constant progression has been the result of steady advances in technology, procedural technique, patient selection, periprocedural management and operator experience. Indeed, TAVR indications are bound to evolve in the next years [9,10].

The objectives of this review were to provide an overview of the current recommendations of TAVR and evaluate the potential avenues for TAVR expansion.

## 2. Current Recommendations

Recommendations on valvular heart disease management were recently updated in both North America and Europe [11,12]. As expected, TAVR has gained importance on the management of severe AS compared to prior versions of the guidelines. The indications for invasive management of AS according to different clinical scenarios are detailed in the Table 1, and the echocardiographic criteria to assess the severity of AS are summarized in Table 2. Briefly, both guidelines recommend an intervention in symptomatic severe AS and in symptomatic and asymptomatic severe low-flow low-gradient AS with reduced left ventricular ejection fraction (LVEF) defined as a LVEF < 50%. In patients with symptomatic low flow low-gradient severe AS and preserved LVEF, the intervention is deemed a Class 1 and IIa recommendation in the US and European guidelines, respectively. In patients with severe asymptomatic AS and preserved LVEF, intervention should be considered in specific clinical scenarios, for example: in the presence of exertional symptoms on stress tests or reduced exercise tolerance, very severe AS, increased serum B-type natriuretic peptide levels (3× over the normal values) or rapidly progressive stenosis (Table 1—Class IIb in both guidelines) [13,14,15,16]. This implies that there is still some room for “watchful waiting” in asymptomatic severe AS. Finally, there is no recommendation for early invasive management of moderate AS outside of SAVR when the patient undergoes cardiac surgery for another reason. An obvious gap in evidence remains for asymptomatic severe AS that falls outside specific clinical scenarios (mainly reduced LVEF) and early invasive management of moderate AS. The feasibility and relative safety of TAVR would provide the substrate to expand the interventional indications of AS management in these scenarios.

**Figure 1:**
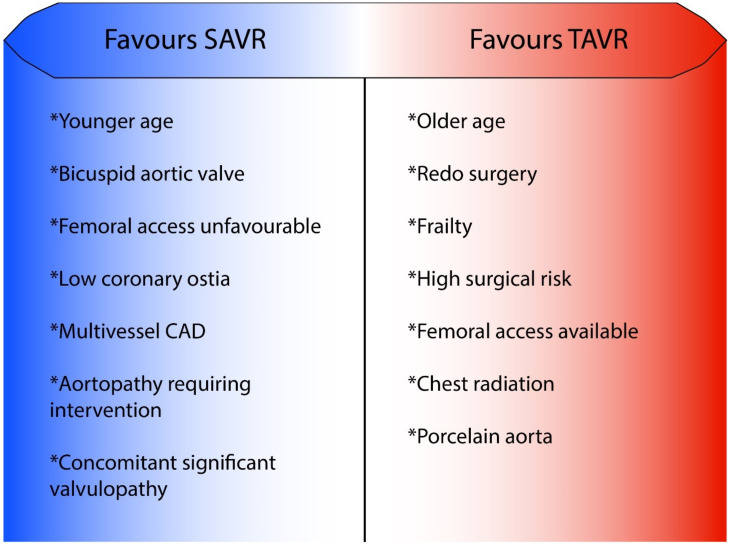
Clinical characteristics in favor of surgical or transcatheter aortic valve replacement. CAD, coronary artery disease; TAVR, transcatheter aortic valve replacement; SAVR, surgical aortic valve replacement.

The indications of TAVR vs. SAVR for the management of AS when a bioprosthesis is required differ slightly between North American and European guidelines. In the North American guidelines, transfemoral TAVR compared to SAVR can be considered in patients over 65 years old, and TAVR is favored in patients over 80 years old or with a life expectancy under 10 years (Figure 1). In the European Society of Cardiology guidelines, TAVR should be considered in low-risk patients over 75 years old, mainly because of concerns on valve durability in addition to the low number of young patients included in low-risk trials [6,7]. Despite this conservative approach in the European guidelines, both scientific societies converge on the fact that transfemoral TAVR should be performed over SAVR in older and higher-risk patients. The presence of a bicuspid aortic valve, rheumatic heart disease, extreme annulus size, other cardiac conditions (e.g., concomitant mitral regurgitation or severe coronary artery disease) as well as unsuitable anatomical characteristics for transfemoral TAVR (e.g., low coronary ostia) should favor SAVR if feasible. Conversely, previous cardiac surgery, porcelain aorta, failed aortic bioprosthesis, severe lung, liver or renal diseases tip the scale toward TAVR. Finally, nontransfemoral TAVR is recommended be restricted to inoperable patients, keeping in mind that palliative care is always an option if life expectancy with a good quality of life is expected to be <12 months. In conclusion, except in young, low-risk patients, the possibility of transfemoral TAVR should always be explored and discussed within the heart team and with the patient. The TAVR vs. SAVR decision should be taken by an experienced heart team considering local expertise and healthcare resources, age, life expectancy, comorbidities, associated cardiac conditions, anatomical characteristics and patient preference.

## 3. Where Are We Going?

The current guidelines outline the body of evidence around interventional AS management and give insights on future avenues to expand TAVR indications. There are two main possibilities to broaden TAVR indications: expanding the indications for invasive management of AS, and choosing TAVR over SAVR when aortic valve replacement with a bioprosthesis is indicated.

### 3.1. Expanding the Indications of Interventional Management of AS

**Figure 2:**
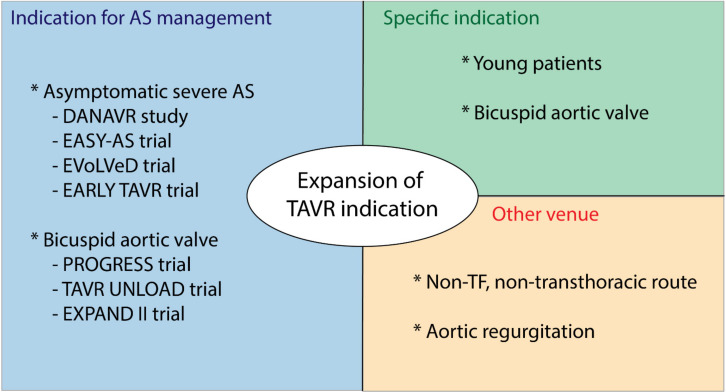
Future direction in TAVR indication; AS, aortic stenosis; TF, transfemoral.

The excellent efficacy and safety results of transfemoral TAVR in all surgical-risk categories has led to an increasing interest in expanding the indications of invasive management of AS toward severe asymptomatic AS and moderate AS (Figure 2).

**Asymptomatic severe aortic stenosis.** Asymptomatic severe AS is a common entity in clinical practice, with 25 to 50% of patients with severe AS reporting no symptoms at the time of diagnosis [17,18]. As previously mentioned (Table 2), aortic valve replacement is recommended only in selected patients with asymptomatic severe AS, namely, those with very severe (transvalvular velocity ≥ 4.5 m/s) or rapidly progressive AS, elevated cardiac biomarkers or falsely asymptomatic AS (with exertional symptoms). Therefore, current guidelines recommend watchful waiting in the vast majority of patients until symptoms or left ventricular systolic dysfunction occur.

There are two major issues with this strategy. First, confirming the lack of AS-related symptoms may be challenging. They could be masked by a sedentary lifestyle or falsely attributed to other comorbidities or old age. Consequently, a systematic stress test is recommended to unmask falsely asymptomatic AS and exertional symptoms. However, it could still be uninformative or misleading if exercise capacity is too low [19,20]. The second issue is that this consensus is based on the balance between the estimated risk of sudden death in this population (between 1 and 1.5% per year) vs. the periprocedural mortality of SAVR or TAVR [21,22]. The current low periprocedural mortality rates for SAVR and TAVR in low-risk patients could tip the balance toward an earlier intervention in this population.

Several reports have advocated for early aortic valve replacement compared to conservative care [16,22]. A meta-analysis including four observational studies that pooled data from 2486 patients (552 [21%] undergoing aortic valve replacement and 1964 [79%] in the conservative approach group) concluded that early aortic valve replacement was associated with a reduced risk of all-cause death (risk ratio 0.29, 95% confidence interval 0.17–0.51) [22]. Two recent randomized trials confirmed these results. First, Kang et al. reported that early SAVR was associated with a lower rate of cardiovascular death (including operative mortality) compared to conservative management after a mean follow-up of ~6 years in 145 patients with very severe asymptomatic aortic stenosis [14]. The AVATAR trial (*Aortic Valve Replacement Versus Conservative Treatment in Asymptomatic Severe Aortic Stenosis*), which included 157 patients and required a negative exercise test to confirm asymptomatic AS, showed that early SAVR reduced the composite outcome of all-cause death, myocardial infarction, stroke or unplanned hospitalization for heart failure compared to conservative treatment [23]. While these results would support early invasive management of severe asymptomatic AS, they should not be extrapolated to TAVR. Indeed, in the trial by Kang et al., patients were at a very low risk (mean EuroSCORE II 0.9%), with a high rate of bicuspid valves (54%), and 50% received a mechanical valve, whereas in the AVATAR study, 53% of patients undergoing SAVR were treated with a mechanical valve [14,23].

Several trials are underway to evaluate early invasive management of asymptomatic severe AS in patients with preserved LVEF (>50%). Details of ongoing trials in asymptomatic severe AS are shown in Table 3. Two trials include both SAVR and TAVR in the interventional group: the EASY-AS trial (*The Early Valve Replacement in Severe ASYmptomatic Aortic Stenosis Study*—NCT04204915) and the DANAVR study (*Danish National Randomized Study on Early Aortic Valve Replacement in Patients with Asymptomatic Severe Aortic Stenosis*—NCT03972644). The EASY-AS trial is planning to randomize 2844 patients with asymptomatic severe AS without left ventricular dysfunction between watchful waiting and early aortic valve replacement (either with SAVR or TAVR) with a 5-year follow-up. The primary outcome is a composite of all-cause death and hospitalization for heart failure. The DANAVR study is randomizing 1700 patients with asymptomatic severe AS and persevered LVEF (but with subclinical signs of left ventricular dysfunction) to early aortic valve replacement (SAVR or TAVR) or medical treatment, with all-cause mortality as the primary outcome. The EVoLVeD trial (*Early Valve Replacement Guided by Biomarkers of LV Decompensation in Asymptomatic Patients with Severe AS*—NCT03094143), which will include 1000 patients participating in the EASY-AS trial, will specifically evaluate early aortic valve replacement compared to watchful waiting in patients with mid left ventricular fibrosis as assessed by magnetic resonance imaging [24]. Two additional trials are focusing specifically on TAVR in asymptomatic severe AS. The EXPAND I trial (NCT04639258), is a single-arm feasibility study with the self-expandable Evolut valve that will include 75 patients. The EARLY-TAVR trial (*Evaluation of TAVR Compared to Surveillance for Patients with Asymptomatic Severe Aortic Stenosis*—NCT03042104) is a randomized trial that will include 1109 patients over 65 years old, with asymptomatic severe AS confirmed or not with a negative treadmill test [25]. It will compare early transfemoral TAVR versus the standard of care with a composite primary outcome of all-cause death, all-stroke and unplanned cardiovascular hospitalization at 2 years.

**Moderate aortic stenosis**. Moderate AS is not a rare finding in old patients [26]. According to both US and European guidelines, aortic valve replacement should be considered in patients with moderate AS undergoing cardiac surgery for other reasons (class IIa in both guidelines), and invasive management is not indicated in patients with isolated, moderate AS [11,12]. However, evidence from observational studies suggest that moderate AS has a negative impact on clinical outcomes, especially in patients with reduced ejection fraction [27,28]. Whereas afterload reduction with medical therapy (with beta blockers, angiotensin-converting enzyme inhibitors or angiotensin II receptor blockers, angiotensin receptor neprilysin inhibitors, aldosterone agonists) is an essential part of the management of heart failure with reduced ejection fraction, only aortic valve replacement can improve the valvular component of afterload secondary to moderate AS. As such, significant improvements in clinical outcomes have been reported after both SAVR and TAVR in observational studies in these patients. In a retrospective analysis including 1090 patients with moderate AS (mean aortic gradient ≥ 25 and <40 mmHg) and reduced ejection fraction (<50%), 287 (26%) patients underwent SAVR, which was associated with an improvement in 5-year survival (Hazard Ratio (HR) 0.68, 95% confidence interval (CI) 0.52–0.90, *p* = 0.007) [29]. In a recent retrospective analysis of 262 patients with moderate AS (defined by aortic valve area > 1.0 and <1.5 cm^2^ and peak velocity > 2 and <4 m/s) and reduced LVEF (<50%), 44 underwent aortic valve replacement (15 TAVR). After a median follow-up of 2.9 ± 2.2 years (median time to aortic valve replacement of 10.9 ± 16 months), aortic valve replacement was associated with improved survival (HR 0.59, 95% CI 0.35–0.98, *p* = 0.04). Interestingly, TAVR but not SAVR was associated with improved survival (HR 0.43, 95% CI 0.18–1.00, *p* = 0.05) [30].

**Figure 3:**
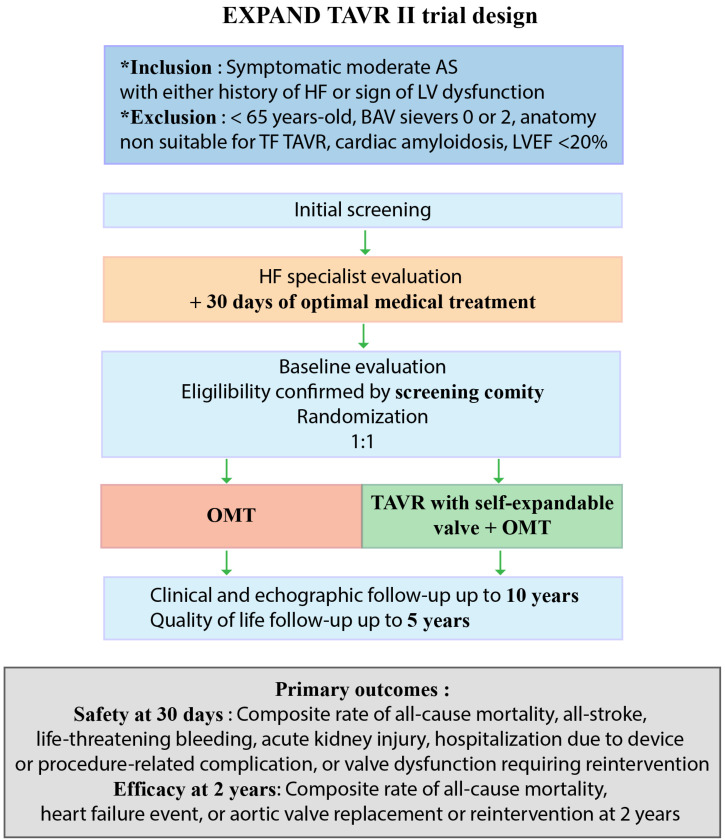
EXPAND TAVR II trial study design; AS, aortic stenosis; BAV, bicuspid aortic valve; HF, heart failure; OMT, optimal medical therapy; TAVR, transcatheter aortic valve replacement; TF, transfemoral.

The hypothesis that TAVR might improve outcomes in patients with moderate AS and heart failure with reduced LVEF is currently being evaluated in several randomized trials (Table 3). The TAVR-UNLOAD trial (*Transcatheter Aortic Valve Replacement to UNload the Left Ventricle in Patients with ADvanced Heart Failure*—NCT02661451) is an open-label, randomized trial that will include 300 patients with heart failure, reduced LVEF and moderate AS which are symptomatic despite optimal medical therapy [31]. The two treatment arms (randomized in a 1:1 fashion) are optimal medical therapy plus transfemoral TAVR with the Edwards SAPIEN 3 valve or optimal medical therapy alone. The primary outcome is the hierarchical occurrence of all-cause death, disabling stroke, hospitalizations related to heart failure, symptomatic aortic valve disease or nondisabling stroke, and the change in the Kansas City Cardiomyopathy Questionnaire at one year [31]. The PROGRESS trial (*A Prospective, Randomized, Controlled Trial to Assess the Management of Moderate Aortic Stenosis by Clinical Surveillance or Transcatheter Aortic Valve Replacement*—NCT04889872) is an open-label, randomized trial that will include 750 patients over 64 years of age, with moderate AS with either evidence of cardiac dysfunction or AS-related symptoms. In these patients, the association of optimal medical therapy and transfemoral TAVR with the Edwards SAPIEN 3 valve will be compared to optimal medical therapy alone. The primary outcome is a composite of all-cause death, stroke or unplanned cardiovascular hospitalization at 2 years. Finally, the EXPAND II trial (NCT05149755) recently started (Figure 3). It will compare optimal medical therapy to TAVR with the self-expandable Evolut PRO+ valve in addition to optimal medical therapy in patients with symptomatic moderate AS and one of the following: at least one heart failure decompensation episode in the past year, elevated NTproBNP levels, reduced longitudinal strain (≤15%) or E/e’ over 14. It will therefore include patients with reduced and preserved LVEF. The primary outcome is a composite of all-cause mortality, heart failure or aortic valve replacement at 2 years. The trial will include 650 patients.

Of note, unlike asymptomatic severe AS, trials in moderate AS and heart failure with reduced LVEF will evaluate TAVR only (and not SAVR). This could be explained by the higher complication rate associated with open-heart surgery compared to TAVR in this population. Interestingly, if these trials demonstrate a benefit in early intervention with TAVR, it would be the first specific indication for TAVR (without SAVR).

Finally, a recent report also showed an association between moderate AS—irrespective of LVEF—and poorer outcomes compared to patients with mild or no AS. While most comorbidities were not accounted for in this study, some of these patients (particularly those with a more rapid progression of AS) could benefit from early aortic valve replacement (either TAVR or SAVR) [28]. It is most likely that moderate AS will gain in granularity in the coming years and that early invasive management will be proposed to some patients.

### 3.2. Choosing TAVR over SAVR When Aortic Valve Replacement with a Bioprosthesis Is Indicated

Despite the excellent results of TAVR vs. SAVR in the whole surgical-risk spectrum, there are many clinical situations where SAVR would be favored over TAVR [11,12]. Two issues in particular are critical for the future development of TAVR, especially in low-risk patients: the extension of TAVR to younger patients and patients with a bicuspid aortic valve (BAV). Finally, we will also review the future of valve-in-valve (ViV) procedures.

**TAVR in young patients.** Considering that TAVR has shown its efficacy compared to SAVR in low-risk patients, surgical risk is not a limitation for choosing TAVR over SAVR anymore when biological aortic valve replacement is indicated [6,7]. In low-risk patients, the main limiting factor is now young age. Indeed, mean age was relatively high in the PARTNER 3 (*Placement of Aortic Transcatheter Valves*) (73 ± 6 years) and Evolut Low-Risk (74 ± 6 years) trials.

One of the most important concerns raised by extending TAVR to low-risk, younger patients is valve durability. In the field of surgical bioprosthetic valves, it is known that a younger age at implantation is associated with accelerated structural valve deterioration, and it is likely that such findings would also apply to TAVR [32]. As a relatively young technology, long-term data on TAVR durability remains scarce, particularly with newer generation devices. Data from current reports are reassuring but insufficient: in the 5-year results of the PARTNER 1A trial, no significant structural valve deterioration was noticed in both SAVR and TAVR groups, albeit the death rate was over 60% in both groups [33]. In studies with early generation self-expandable valves, significant bioprosthesis failure was reported in 1.4% of cases at 5 years (with a death rate over 50% at 5 years) [34]. In the NOTION (*Nordic Aortic Valve Intervention*) trial, which randomized low-surgical-risk patients to TAVR vs. SAVR, no significant differences in the risk of bioprosthetic valve failure were observed between groups at 8-year follow-up (8.7% vs. 10.5% in the TAVR and SAVR groups, respectively, *p* = 0.61), and TAVR patients had less structural valve deterioration (13.9% vs. 28.3% in the TAVR and SAVR groups, respectively, *p* = 0.002) [35]. Finally, observational data on 241 TAVR patients with a follow-up up to 10 years (64% of self-expandable valves) showed severe structural valve deterioration in 0.4% and moderate structural valve deterioration in 1/12 patients [36]. While these data appear to be reassuring, the immortal-time bias remains a major issue given the high mortality rate (i.e., only patients alive can be assessed for structural valve deterioration, which might have been missed because of early death). There is a need for a consensual definition of structural valve deterioration so that studies can be compared. The valve academic research consortium 3 (VARC-3) criteria provided a definition for aortic bioprosthesis valve dysfunction, which includes structural valve deterioration (further graded in morphological, moderate haemodynamic or severe haemodynamic valve deterioration) [37]. In the VARC-3 consensus paper, structural valve deterioration is differentiated from valve thrombosis, nonstructural valve dysfunction and endocarditis [37]. Importantly, studies on long-term durability of transcatheter valves should focus on all aspects of aortic bioprosthesis valve dysfunction and not only structural valve deterioration. Long-term data of low-risk trials (with a yearly follow-up of up to 10 years) are eagerly awaited.

Another important issue in lower-risk, younger patients is the rate of permanent pacemaker implantation after TAVR. Compared to SAVR, TAVR has been associated with an increased risk of conduction disturbances and subsequent pacemaker implantation [38]. However, this rate has been highly variable: in the PARTNER 3 trial the rate of pacemaker implantations was similar between SAVR and TAVR with a balloon-expandable valve, but it was significantly higher in the Evolut Low-Risk trial with a self-expandable valve (17.4% vs. 6.1% in the TAVR and SAVR groups, respectively) [6,7]. Some patient characteristics have also been associated with an increased risk of conduction disturbances requiring pacemaker implantation, notably the presence of pre-existing right bundle block branch [39]. Permanent pacemaker implantation has consequences after TAVR, and it has been associated with an increased risk of mortality and heart failure hospitalization at 1 year in high-risk patients [40]. Given the longer lifespan of younger low-risk patients, potential complications of intracardiac leads as well as long-term consequences of permanent pacing and left ventricle desynchronization should be considered. Of note, the rate of permanent pacemaker implantation has decreased in recent TAVR series, likely due to improved valve positioning with lower implantation depth (a more aortic valve implant) [41]. Monitoring permanent pacemaker implantation in young, low-risk patients will be crucial.

Another important issue in these patients is coronary artery disease (CAD) management and coronary access after TAVR. Considering the longer lifespan, the probability of coronary intervention is non-negligible—especially considering the known association between severe AS and coronary artery disease [42]. TAVR valves have a valve stent frame which may impair coronary access, especially when it comes to self-expandable valve systems with a taller stent frame and a supra-annular valve leaflet insertion [42,43,44,45]. As such, coronary access in ST-elevation myocardial infarction in patients with TAVR took longer compared to non-TAVR patients, and up to 4.2% of patients could not undergo coronary revascularization because of coronary ostia cannulation failure [46]. In the RE-ACCESS study (*Reobtain Coronary Ostia Cannulation Beyond Transcatheter Aortic Valve Stent*), 7.7% of unsuccessful coronary canulations were observed after TAVR, mainly in the presence of self-expanding Evolut valves [47]. To overcome these difficulties, Yudi et al. proposed a catheter selection algorithm depending on the type of bioprosthesis [44], as well as the systematic use of six French catheters via a left radial or femoral approach. The use of a guide extension catheter could also help to achieve selective canulation of the coronary artery [44]. Still, the optimal management of CAD prior to TAVR intervention remains unclear [42], with controversial results on the association of CAD and complete revascularization and clinical outcomes after TAVR [48,49]. Several grey area aspects remain regarding the optimal method for assessing CAD and the clinical relevance of percutaneous coronary intervention in the TAVR work-up. Invasive coronary angiogram has been the gold standard to assess CAD in the early experience of TAVR. However, computed coronary angiography, as well as functional evaluation of coronary stenosis, have been evaluated in the TAVR work-up with promising results [42]. Computed coronary angiography can be performed during the mandatory cardiac computed tomography in TAVR, and has an excellent negative predictive value to rule out significant coronary stenosis [50]. It is therefore used as a gatekeeper for invasive coronary angiograms in some centers, especially in low-risk patients with a lower probability of pre-existing CAD [6]. A hemodynamic assessment of CAD, with either invasive fractional flow reserve or computed tomography fractional flow reserve, needs to be further evaluated in the setting of TAVR work-up but might have a role in CAD diagnosis in these patients [51,52]. Several trials are ongoing in this field [42]. Despite these considerations, the optimal extent of coronary revascularization in these patients remains unknown. In patients without severe AS, randomized trials have failed to demonstrate improved outcomes with the invasive management of stable CAD compared to optimal medical therapy [53,54]. Nonetheless, in the ISCHEMIA trial (*International Study of Comparative Health Effectiveness with Medical and Invasive Approaches*), invasive management reduced the rate of type 1 myocardial infarction compared to optimal medical therapy—which could prevent the life-threatening situation of extremely difficult or impossible coronary reaccess in ST-elevation myocardial infarction after TAVR [55]. On the other hand, extensive percutaneous revascularization increases the risk of stent failure and similar reaccess issues after TAVR. Given the current clinical evidence, guidelines advocate for revascularization in cases with significant stenosis in the proximal segment of coronary arteries prior to TAVR regardless of symptoms (Class IIa) [12,56].

**TAVR in bicuspid aortic valves.** With a prevalence of 1 to 2% in the general population, BAV is the most common congenital heart disease, and not only in younger patients: approximately 25% of patients over 80 years old referred to SAVR had BAV [57].

BAV patients were excluded from most TAVR vs. SAVR randomized controlled trials because of potential anatomical challenges that could increase the risk of periprocedural complications and procedural failure [6,7]. These challenges are now well known [58]. First, BAV is associated with a higher calcium burden which can be eccentric, leading to an increased risk of complications such as the suboptimal expansion of the valve, or aortic annulus injury [59]. Indeed, in a recent multicenter registry, calcified raphe and excess leaflet calcification (defined as more than median calcium volume) were independent predictors of 2-year mortality after TAVR in BAV, with an increased risk if both characteristics were present [60]. Second, BAV is frequently associated with a more elliptical sinus of Valsalva which could contribute to valve prosthesis underexpansion, with a potential negative impact on valve durability and thrombosis [61]. Furthermore, aortic annuli in BAV are usually larger compared to tricuspid aortic valve annulus, and some patients might fall outside the annulus dimensions covered by TAVR valves. Third, the aorta is commonly more horizontal in BAV patients. Finally, aortopathy is commonly associated with BAV, with approximately 25% of BAV patients undergoing replacement of the ascending aorta at the time of SAVR [62]. However, these challenges can be managed by a careful preoperative assessment.

The first results of TAVR in BAV patients were published with the early iterations of TAVR devices, with relatively high rates of procedural complications [63]. In 2017, a matched comparison of 546 BAV patients vs. tricuspid aortic valve patients treated with TAVR showed similar mortality rates at a 2-year follow-up but a higher rate of surgical conversion in the BAV group (2% vs. 0.2% in BAV and tricuspid aortic valve patients, respectively, *p* = 0.006). A similar analysis was performed comparing 932 matched pairs (BAV and tricuspid aortic valve) that underwent TAVR with self-expanding valves in the TVT (*transcatheter valve therapy*) registry with similar rates of all-cause mortality at one year [64]. Overall, observational studies have shown that TAVR is a reasonable option in selected BAV patients.

There is, nonetheless, some room for improvement. First, identifying risk factors associated with reduced device success and poorer outcomes is critical [60]. Second, a possible increased risk of ischemic stroke post-TAVR in BAV patients remains a concern, potentially related to the higher calcium burden of the native valve [65]. An analysis of 204 consecutive patients (83 BAV) that underwent systematic cerebral magnetic resonance imaging post-TAVR showed a higher number of new lesions (*p* = 0.008) and a larger volume per lesion (*p* = 0.04) in BAV patients, though the rate of clinically apparent strokes remained similar in both groups (2.4% vs. 1.7%, in the BAV and tricuspid aortic valve groups, respectively, *p* = 0.70) [66]. The use of cerebral protection devices might play an important role in this setting. Third, the evolution of aortopathy, especially in mid-range aortic dilatation, could be a concern. Correcting AS might prevent aortopathy progression (by reducing aortic wall shear stress), but more data are needed. Finally, concerns about valve durability would also apply to a significant proportion of patients with BAV.

There are two ongoing clinical trials in the field of TAVR and BAV. The first one is a prospective single-arm study with the self-expandable Evolut Pro device (Medtronic) in low-risk patients with BAV that will include 150 patients with a follow-up of 10 years. This study will provide critical information on this population as well as on long-term valve durability. The second one is a multicenter, open-label, noninferiority randomized trial that will compare SAVR to TAVR in 300 patients with BAV and intermediate surgical risk in China (NCT03163329). Finally, the results from the imbedded registry of BAV patients in the PARTNER 3 trial, with the last generation balloon-expandable valve, are expected.

Overall, despite being excluded from randomized clinical trials, TAVR provides an efficient solution for selected patients (i.e., with favorable anatomy) with BAV, especially in patients with a high surgical risk. Improvement in patient selection and growing clinical evidence will likely support TAVR as an option for BAV patients with AS in future guideline iterations, initially in patients with increased surgical risk and probably in lower-risk patients in the near future.

**Valve-in-valve TAVR.** ViV TAVR is now commonly performed in patients with failed surgical bioprosthesis, accounting for ~7% of procedures in the transcatheter valve therapeutics registry in 2019 [67]. A large amount of observational data supports the safety and efficacy of ViV TAVR [68,69,70]. Compared to TAVR in native annulus, ViV TAVR has been associated with lower rates of paravalvular leaks, stroke or new permanent pacemaker implantation, but higher residual gradients and higher rates of coronary occlusion [70,71,72]. As such, ViV TAVR requires careful assessment by the heart team for both coronary occlusion risk and patient–prosthesis mismatch (especially in patients with small bioprosthesis) [73,74]. Two specific techniques have been developed to improve or prevent these complications: bioprosthetic valve fracture and the BASILICA technique (*bioprosthetic or native aortic scallop intentional laceration to prevent iatrogenic coronary artery obstruction*). Bioprosthetic valve fracture aims to reduce patient–prothesis mismatch by fracturing the surgical sewing ring of the failed bioprosthesis using a noncompliant valvuloplasty balloon [75]. This leads to a better expansion of the transcatheter valve and an increase in the effective orifice area following ViV TAVR [75,76]. However, the long-term impact of valve fracture on clinical outcomes and TAVR valve durability needs to be further evaluated [77]. The BASILICA is an interventional technique that aims to lacerate valvular leaflets (native or of the failed bioprosthesis) to prevent coronary obstruction [78]. This technique would allow to perform TAVR in otherwise ineligible patients (because of the prohibitive risk of valve leaflet-induced coronary obstruction) [79]. Still, the BASILICA technique is a demanding and complex procedure. Another rising issue is TAVR-in-TAVR procedures for a failed TAVR valve. The worldwide experience remains limited but the same pitfalls as ViV TAVR are expected: coronary access and patient–prothesis mismatch [80,81]. Nonetheless, observational studies suggest good feasibility and short-term outcomes [81], and we will most likely see specific techniques/devices to adapt to these situations [82].

Still, patients with small bioprosthesis are at a high risk of patient–prothesis mismatch after ViV TAVR, which may be associated with a lower survival rate [72]. A randomized comparison between redo-SAVR and ViV TAVR is eagerly needed in these patients, and redo-SAVR should be considered when the risk of patient–prothesis mismatch is too high. Despite this risk, a significant number of redo patients exhibit a prohibitive surgical risk and will undergo ViV TAVR. Bioprosthetic valve fractures and supra-annular transcatheter heart valves might reduce postprocedural gradients and patient–prothesis mismatch [83]. Results from the LYTEN trial (*Comparison of the Balloon-Expandable Edwards Valve and Self-Expandable CoreValve Evolut R or Evolut PRO System for the Treatment of Small, Severely Dysfunctional Surgical Aortic Bioprotheses*—*NCT03520101*) that randomized balloon-expandable vs. self-expandable transcatheter valves in patients with small degenerated surgical bioprosthesis will provide more insights on valve hemodynamic differences between valve types in these patients.

Nonetheless, the good results of ViV TAVR (and, to some extent, TAVR-in-TAVR) have an impact on the long-term management of AS, especially in young patients, and the surgical management of AS should be performed with the planification of a subsequent potential ViV TAVR. Patients with small aortic annulus require thorough heart team discussions to plan for subsequent bioprosthesis failure (either surgical or transcatheter) to ultimately mitigate the risk of patient–prothesis mismatch.

### 3.3. Other Venues to Expand TAVR Indication

**Nontransfemoral access TAVR.** Transfemoral TAVR has been established as the default access for TAVR; however, a proportion of TAVR candidates are not eligible for transfemoral access, either because of peripheral arteriopathy, tortuosity, severe calcification or small vascular access. In contemporary registries, alternative access TAVR represents up to 15% of cases [84,85].

According to both European and US guidelines, patients without favorable transfemoral access should be evaluated for SAVR, and those with nontransfemoral access should be considered only in prohibitive surgical-risk patients unsuitable for transfemoral TAVR (Class IIb Level of evidence C) [11,12]. However, alongside the progress of transfemoral TAVR, improvements have also been made in nontransfemoral TAVR. First, nontransfemoral TAVR also benefits from device enhancements (and associated clinical success) as well as delivery sheath improvement. Second, transthoracic (transaortic or transapical) access has been dropped in favor of less-invasive transarterial accesses such as trans-subclavian or transcarotid [86,87].

Historically, nontransfemoral TAVR was limited to the transthoracic route and was associated with an increased risk of complications compared to transfemoral TAVR in the PARTNER (Placement of Aortic Transcatheter Valves) 1 and 2 trials and observational studies [4,88]. Overall, transthoracic access negated the clinical benefit of TAVR vs. SAVR in the PARTNER 2 trial [4]. Since then, transthoracic routes have been in steep decline at the expense of less-invasive (without thoracotomy) accesses, namely, trans-subclavian/trans-axillary and trans-carotid routes [89]. Despite being performed in sicker and higher risk patients, observational data suggest that TAVR from these alternative transarterial accesses is associated with similar outcomes compared with transfemoral TAVR [89,90]. In the FRANCE TAVI registry (*French Transcatheter Aortic Valve Implantation*), 1616/21,611 (7.5%) procedures were performed by a trans-subclavian or transcarotid access route (3.2% and 4.2% for trans-subclavian and transcarotid, respectively). When these patients were matched to the transfemoral cohort, no differences in the rate of periprocedural complications and death were found (including strokes). Compared to transfemoral access, alternative access reduced the rate of major vascular complications (odds ratio [OR] 0.45; 95% CI 0.21–0.93, *p* = 0.032) and unplanned vascular repairs (OR 0.41; 95% CI 0.29–0.59, *p* < 0.001) after TAVR. This can be explained by the surgical access of trans-subclavian and transcarotid routes: major bleeding that would categorize as a vascular complication is less likely when the artery is surgically exposed and secured. Nonetheless, procedural results were similar between trans-subclavian or transcarotid access and transfemoral access after a propensity-matching analysis. Interestingly, high-volume centers were more likely to perform alternative access TAVR (OR 1.63, 95% CI 1.33–2.01), which reflects the clinical need for alternative routes in highly specialized centres. These results were similar in other registries, showing a high safety profile of transcarotid and trans-subclavian access for TAVR [90,91].

With reassuring observational data and gained experience, nontransfemoral (and nontransthoracic) TAVR could be further evaluated in randomized trials, following the initial path of TAVR: first being compared to SAVR in inoperable or high surgical risk patients with a nonfavorable transfemoral route. Associated with increased observational evidence, this would expand the TAVR indication.

**TAVR for severe aortic regurgitation.** In patients with severe native aortic regurgitation (AR), SAVR is indicated in the presence of symptoms, reduced LVEF or left ventricular enlargement [11,12]. AR is a degenerative disease in most cases, and it is therefore frequently associated with older age and comorbidities. As such, only 21.8% of patients with LVEF between 30 and 50% and 2.7% of those with LVEF < 30% underwent SAVR in the Euro Heart Survey in 2003 [92], reflecting an unmet need for nonsurgical interventional management in the context of severe native AR.

Evidence on the use of TAVR for treating severe native AR has been limited to observational data in small studies including selected patients. Patients treated with TAVR for severe native AR were carefully evaluated by the heart team of each center to ensure that annulus size was small enough to provide consequent oversize and prevent valve embolization. Keeping that in mind, a meta-analysis published in 2016 including 237 patients (79% self-expandable valves) reported that device success was achieved in 74 to 100% and 17 patients (7%) required the implantation of a second valve [93]. These results seemed to improve over time with the use of a newer generation devices and increasing operator experience. Indeed, in 331 patients with severe native AR treated with TAVR (119 early generation and 212 new-generation devices), device success rate was 81.1% with new-generation devices vs. 61.3% with older devices (*p* < 0.001). However, the incidence of second valve implantation remained high: 12.7% with new-generation devices (and 24.4% with older devices).

Percutaneous interventional management of severe native AR remains an unsolved issue. The JenaValve (Trilogy; JenaValve Technology) is a dedicated TAVR valve for severe native AR currently being tested [94]. It is delivered through transfemoral access and is anchored by clipping the aortic cusps—preventing device migration. Initial single-center experience with 11 patients showed good results with no mortality or stroke at 30 days, but a rate of pacemaker implantation of 36.4%. Reduction in AR was significant, with only one patient with mild paravalvular leak [95]. The JenaValve is currently being tested in the ALIGN-AR pivotal trial (NCT04415047), a single-arm study including 180 high surgical risk patients with severe symptomatic AR. Other devices are being studied, such as the J-Valve system (JC Medical) and the Helio transcatheter aortic dock, that could provide anchoring for a balloon-expandable valve [96,97]. Until the validation of dedicated devices, TAVR will remain an off-label indication in selected patients.

## 4. Conclusions

The expansion of TAVR has been relentless in the past decade, and the momentum remains. Given its excellent efficacy and safety results, TAVR has pushed the boundaries of invasive management of AS, with several randomized trials exploring early intervention in asymptomatic severe AS with preserved LVEF and in moderate symptomatic AS. On the other hand, real-life evidence and growing experience will help to broaden TAVR indications over SAVR in selected patients with specific clinical situations such as BAV, AR or younger age. Additionally, the exact role of nontransfemoral (nontransthoracic) TAVR is yet to be determined and could further contribute to extend TAVR indications.

## Figures and Tables

**Table 1:** Indication for invasive management of aortic stenosis.

	AHA Guidelines		ESC Guidelines	
*Indication for Intervention*	Class	Level of Evidence	Class	Level of Evidence
**Symptomatic patients**				
High-gradient severe AS	1	A	I	B
Low-flow low-gradient severe AS with reduced LVEF (<50%)	1	B		
With contractile reserve	-	-	I	B
Without contractile reserve	-	-	IIa	C
Low-flow low-gradient severe AS with preserved LVEF if symptoms related to AS	1	B	IIa	C
**Asymptomatic patients**				
Severe AS and reduced LVEF (<50%)	1	B	I	B
Severe high-gradient AS with exertional symptoms	-	-	I	C
Severe AS with sustain fall in BP	2a	B	IIa	C
Severe AS with decreased exercice tolerance	2a	B		
Very severe AS	2a	B	IIa	B
Severe AS with low procedural risk and: - Vmax progression ≥ 0.3 m/s/year - Elevated biomarkers (BNP > 3× normal)	2a	B	IIa	B
Severe high-gradient AS with a progressive decrease in LVEF on at least 3 serial TTE < 60%	2b	B	-	-
**Other cardiac surgery**				
Severe AS	1	B	I	C
Moderate AS	2b	C	IIa	C

AS, aortic stenosis; BNP, B-natriuretic peptide; BP, blood pressure; LVEF, left ventricular ejection fraction; TTE, transthoracic echocardiography

**Table 2:** Echocardiographic cut-off of aortic stenosis.

	Maximum Velocity	Mean Transaortic Gradient	Aortic Valve Area	LVEF	Stroke Volume Indexed	Other
Severe high-gradient AS	≥4 m/s	≥40 mmH	Irrespective	Irrespective	-	Eliminate high flow status
Very severe AS	≥5 m/s	≥60 mmHg	Irrespective	Irrespective	-	Eliminate high flow status
Low-flow low-gradient severe AS with reduced LVEF	<4 m/s	<40 mmHg	≤1 cm^2^ ≤0.6 cm^2^/m^2^	LVEF < 50%	≤35 mL/m^2^	
Low-flow low-gradient severe AS with persevered LVEF	<4 m/s	<40 mmHg	≤1 cm^2^ ≤0.6 cm^2^/m^2^	LVEF ≥ 50%	≤35 mL/m^2^	Measured in normotensive patients (SBP < 140 mmHg)
Moderate AS	-	20 to 40 mmHg	1.0–1.5 cm^2^ *	Irrespective	-	In normal flow condition

AS, aortic stenosis; LVEF, left ventricular ejection fraction; SBP, systolic blood pressure. * Or > 1.0 cm^2^ at stress echo if reduced LVEF.

**Table 3:** Current trials aiming to expand TAVR indication.

Trial Name	NCT	Design	Population	Inclusion Criteria	Primary Outcome	Estimated Completion Date
**Asymptomatic severe aortic stenosis**						
DANAVR (*Danish National Randomized Study on Early Aortic Valve Replacement in Patients With Asymptomatic Severe Aortic Stenosis*)	NCT03972644	Open randomized trial; Watchful waiting vs. SAVR or TAVR	1700 patients	Asymptomatic severe AS with preserved LVEF but subclinical sign of LV dysfunction	All-cause mortality (5-year time frame)	September 2029
EASY-AS (*The Early Valve Replacement in Severe ASYmptomatic Aortic Stenosis Study*)	NCT04204915	Open randomized trial; Watchful waiting vs. TAVR or SAVR	2844 patients	Asymptomatic severe AS with preserved LVEF	Composite outcome of all-cause death and hospitalization for heart failure after 663 events	October 2029
EVoLVeD (*Early Valve Replacement Guided by Biomarkers of LV Decompensation in Asymptomatic Patients with Severe AS*)	NCT03094143	Associated with EASY-AS Randomized early intervention according to presence of mid-LV fibrosis in MRI	1000 patients	Asymptomatic severe AS with preserved LVEF	Composite of all-cause mortality or unplanned aortic stenosis-related hospitalization up until study completion (estimated 2.75 years of follow-up)	October 2024
EARLY-TAVR (*Evaluation of TAVR Compared to Surveillance for Patients With Asymptomatic Severe Aortic Stenosis*)	NCT03042104	Open randomized trial; TAVR vs. watchful waiting	901 patients	Asymptomatic severe AS with preserved LVEF and age ≥ 65 years old	All-cause death, all stroke, and unplanned cardiovascular hospitalization at 2 years	March 2024
EXPAND I—Feasibility study	NCT04639258	Single group trial	75 patients	Asymptomatic severe AS with preserved LVEF over 65 years old	All-cause and cardiovascular mortality at 30 days	July 2022
**Moderate aortic stenosis**						
Evolut™ EXPAND TAVR II Pivotal Trial	NCT05149755	Open randomized trial; TAVR and OMT vs. OMT	650 patients	Symptomatic moderate AS with either HF in the past year or elevated cardiac biomarkers or reduced longitudinal strain (≤15%) or elevated LV filling pressures. Age ≥ 65 years old.	Composite of all-cause mortality, all-stroke, life-threatening bleeding, acute kidney injury, hospitalization due to device or procedure-related complication, or valve dysfunction requiring reintervention at 30 days	February 2026
PROGRESS (*A Prospective, Randomized, Controlled Trial to Assess the Management of Moderate Aortic Stenosis by Clinical Surveillance or Transcatheter Aortic Valve Replacement*)	NCT04889872	Open randomized trial; TAVR and OMT vs. OMT	750 patients	Moderate AS with evidence of cardiac dysfunction or symptoms and age ≥ 65 years old	Composite of death, stroke, and unplanned cardiovascular hospitalization at 2 years	June 2029
TAVR UNLOAD (*Transcatheter Aortic Valve Replacement to UNload the Left Ventricle in Patients With ADvanced Heart Failure*)	NCT02661451	Open randomized trial; TAVR and OMT vs. OMT	300 patients	Symptomatic moderate AS with LVEF < 50%	All-cause death at 1 year	March 2023
**Other trials**						
NOTION-II (*Comparison of Transcatheter Versus Surgical Aortic Valve Replacement in Younger Low Surgical Risk Patients With Severe Aortic Stenosis*)	NCT02825134	Open randomized trial; TAVR vs. SAVR	372 patients	Symptomatic severe AS low-risk (STS-PROM < 4%) patients suitable for transfemoral TAVR and <75 years old	Composite of all-cause mortality, stroke and device-related rehospitalization at 1 year	December 2029

AS, aortic stenosis; HF, heart failure; LV, left ventricle; LVEF, left ventricle ejection fraction; OMT, optimal medical treatment; SAVR, surgical aortic valve replacement; STS-PROM, Society of Thoracic Surgeons predicted risk of mortality; TAVR, transcatheter aortic valve replacement.

## Abbreviation

AS	aortic stenosis
BAV	bicuspid aortic valve
LVEF	left ventricular ejection fraction
SAVR	surgical aortic valve replacement
TAVR	transcatheter aortic valve replacement

## References

[1] Cribier A., Eltchaninoff H., Tron C., Bauer F., Agatiello C., Sebagh L., Bash A., Nusimovici D., Litzler P.Y., Bessou J.-P. (2004). Early experience with percutaneous transcatheter implantation of heart valve pros-thesis for the treatment of end-stage inoperable patients with calcific aortic stenosis. J. Am. Coll. Cardiol..

[2] Smith C.R., Leon M.B., Mack M.J., Miller D.C., Moses J.W., Svensson L.G., Tuzcu E.M., Webb J.G., Fontana G.P., Makkar R.R. (2011). Transcatheter versus Surgical Aortic-Valve Replacement in High-Risk Patients. N. Engl. J. Med..

[3] Adams D.H., Popma J.J., Reardon M.J., Yakubov S.J., Coselli J.S., Deeb G.M., Gleason T.G., Buchbinder M., Hermiller J., Kleiman N.S. (2014). Transcatheter Aortic-Valve Replacement with a Self-Expanding Prosthesis. N. Engl. J. Med..

[4] Leon M.B., Smith C.R., Mack M.J., Makkar R.R., Svensson L.G., Kodali S.K., Thourani V.H., Tuzcu E.M., Miller D.C., Herrmann H.C. (2016). Transcatheter or Surgical Aortic-Valve Replacement in Intermediate-Risk Patients. N. Engl. J. Med..

[5] Reardon M.J., Van Mieghem N.M., Popma J.J., Kleiman N.S., Søndergaard L., Mumtaz M., Adams D.H., Deeb G.M., Maini B., Gada H. (2017). Surgical or Transcatheter Aortic-Valve Replacement in Intermediate-Risk Patients. N. Engl. J. Med..

[6] Mack M.J., Leon M.B., Thourani V.H., Makkar R., Kodali S.K., Russo M., Kapadia S.R., Malaisrie S.C., Cohen D.J., Pibarot P. (2019). Transcatheter Aortic-Valve Replacement with a Balloon-Expandable Valve in Low-Risk Patients. N. Engl. J. Med..

[7] Popma J.J., Deeb G.M., Yakubov S.J., Mumtaz M., Gada H., O’Hair D., Bajwa T., Heiser J.C., Merhi W., Kleiman N.S. (2019). Transcatheter Aortic-Valve Replacement with a Self-Expanding Valve in Low-Risk Patients. N. Engl. J. Med..

[8] Bowdish M.E., D’Agostino R.S., Thourani V.H., Schwann T.A., Krohn C., Desai N., Shahian D.M., Fernandez F.G., Badhwar V. (2021). STS Adult Cardiac Surgery Database: 2021 Update on Outcomes, Quality, and Research. Ann. Thorac. Surg..

[9] Thourani V.H., Yadav P.K., Prendergast B. (2022). TAVR Sustains Its Promise in Low-Risk Patients, But the Journey Is Far from Over. J. Am. Coll. Cardiol..

[10] Sá M.P.B., Simonato M., Eynde J.V.D., Cavalcanti L.R.P., Roever L., Bisleri G., Dokollari A., Dvir D., Zhigalov K., Ruhparwar A. (2021). Asymptomatic severe aortic stenosis, bicuspid aortic valves and moderate aortic stenosis in heart failure: New indications for transcatheter aortic valve implantation. Trends Cardiovasc. Med..

[11] Vahanian A., Beyersdorf F., Praz F., Milojevic M., Baldus S., Bauersachs J., Capodanno D., Conradi L., De Bonis M., De Paulis R. (2022). 2021 ESC/EACTS Guidelines for the management of valvular heart disease. Eur. Heart J..

[12] Otto C.M., Nishimura R.A., Bonow R.O., Carabello B.A., Erwin J.P., Gentile F., Jneid H., Krieger E.V., Mack M., McLeod C. (2021). 2020 ACC/AHA Guideline for the Management of Patients with Valvular Heart Disease: Executive Summary: A Report of the American College of Cardiology/American Heart Association Joint Committee on Clinical Practice Guidelines. J. Am. College Cardiol..

[13] Maréchaux S., Hachicha Z., Bellouin A., Dumesnil J.G., Meimoun P., Pasquet A., Bergeron S., Arsenault M., Le Tourneau T., Ennezat P.V. (2010). Usefulness of exercise-stress echocardiography for risk stratification of true asymptomatic patients with aortic valve stenosis. Eur. Heart J..

[14] Garry J.D., Goldman M., Kohlwes J., Sidebotham D., Morrow C.D., Drake D.H., Kang D.-H., Park S.-W. (2020). Early Surgery or Conservative Care for Asymptomatic Aortic Stenosis. N. Engl. J. Med..

[15] Nakatsuma K., Taniguchi T., Morimoto T., Shiomi H., Ando K., Kanamori N., Murata K., Kitai T., Kawase Y., Izumi C. (2018). B-type natriuretic peptide in patients with asymptomatic severe aortic stenosis. Heart.

[16] Taniguchi T., Morimoto T., Shiomi H., Ando K., Kanamori N., Murata K., Kitai T., Kawase Y., Izumi C., Miyake M. (2015). Initial Surgical Versus Conservative Strategies in Patients with Asymptomatic Severe Aortic Stenosis. J. Am. Coll. Cardiol..

[17] Iung B., Delgado V., Rosenhek R., Price S., Prendergast B., Wendler O., De Bonis M., Tribouilloy C., Evangelista A., Bogachev-Prokophiev A. (2019). Contemporary Presentation and Management of Valvular Heart Disease: The EU-RObservational Research Programme Valvular Heart Disease II Survey. Circulation.

[18] Pai R.G., Kapoor N., Bansal R.C., Varadarajan P. (2006). Malignant Natural History of Asymptomatic Severe Aortic Stenosis: Benefit of Aortic Valve Replacement. Ann. Thorac. Surg..

[19] Redfors B., Pibarot P., Gillam L.D., Burkhoff D., Bax J.J., Lindman B., Bonow R.O., O’Gara P.T., Leon M.B., Généreux P. (2017). Stress Testing in Asymptomatic Aortic Stenosis. Circulation.

[20] Pierard L.A., Dulgheru R. (2017). Exercise Testing and Stress Imaging in Aortic Valve Disease. Curr. Treat. Options Cardiovasc. Med..

[21] Pellikka P.A., Sarano M.E., Nishimura R.A., Malouf J.F., Bailey K.R., Scott C.G., Barnes M.E., Tajik A.J. (2005). Outcome of 622 Adults with Asymptomatic, Hemodynamically Significant Aortic Stenosis During Prolonged Follow-Up. Circulation.

[22] Généreux P., Stone G.W., O’Gara P.T., Marquis-Gravel G., Redfors B., Giustino G., Pibarot P., Bax J.J., Bonow R.O., Leon M.B. (2016). Natural History, Diagnostic Approaches, and Therapeutic Strategies for Patients with Asymptomatic Severe Aortic Stenosis. J. Am. Coll. Cardiol..

[23] Banovic M., Putnik S., Penicka M., Doros G., Deja M.A., Kockova R., Kotrc M., Glaveckaite S., Gasparovic H., Pavlovic N. (2022). Aortic Valve Replacement Versus Conservative Treatment in Asymptomatic Severe Aortic Stenosis: The AVATAR Trial. Circulation.

[24] Bing R., Everett R.J., Tuck C., Semple S., Lewis S., Harkess R., Mills N., Treibel T., Prasad S., Greenwood J.P. (2019). Rationale and design of the randomized, controlled Early Valve Replacement Guided by Biomarkers of Left Ventricular Decompensation in Asymptomatic Patients with Severe Aortic Stenosis (EVOLVED) trial. Am. Heart J..

[25] Genereux P. Rationale and Status Update of the EARLY TAVR Trial Asymptomatic Severe AS Patients. Proceedings of the TCT 2017.

[26] Benjamin E.J., Virani S.S., Callaway C.W., Chamberlain A.M., Chang A.R., Cheng S., Chiuve S.E., Cushman M., Delling F.N., Deo R. (2018). Heart Disease and Stroke Statistics-2018 Update: A Report From the American Heart Association. Circulation.

[27] Van Gils L., Clavel M.-A., Vollema E.M., Hahn R.T., Spitzer E., Delgado V., Nazif T., De Jaegere P.P., Geleijnse M.L., Ben-Yehuda O. (2017). Prognostic implications of moderate aortic stenosis in patients with left ventricular systolic dysfunction. J. Am. Coll. Cardiol..

[28] Strange G., Stewart S., Celermajer D., Prior D., Scalia G.M., Marwick T., Ilton M., Joseph M., Codde J., Playford D. (2019). Poor Long-Term Survival in Patients with Moderate Aortic Stenosis. J. Am. Coll. Cardiol..

[29] Samad Z., Vora A.N., Dunning A., Schulte P.J., Shaw L.K., Al-Enezi F., Ersboll M., McGarrah R.W., Vavalle J.P., Shah S.H. (2016). Aortic valve surgery and survival in patients with moderate or severe aortic stenosis and left ventricular dysfunction. Eur. Heart J..

[30] Jean G., Van Mieghem N.M., Gegenava T., van Gils L., Bernard J., Geleijnse M.L., Vollema E.M., El Azzouzi I., Spitzer E., Delgado V. (2021). Moderate Aortic Stenosis in Patients with Heart Failure and Reduced Ejection Fraction. J. Am. Coll. Cardiol..

[31] Spitzer E., Van Mieghem N.M., Pibarot P., Hahn R.T., Kodali S., Maurer M.S., Nazif T., Rodés-Cabau J., Paradis J.-M., Kappetein A.-P. (2016). Rationale and design of the Transcatheter Aortic Valve Replacement to UNload the Left ventricle in patients with ADvanced heart failure (TAVR UNLOAD) trial. Am. Heart J..

[32] Johnston D.R., Soltesz E.G., Vakil N., Rajeswaran J., Roselli E.E., Sabik J.F., Smedira N.G., Svensson L.G., Lytle B.W., Blackstone E.H. (2015). Long-Term Durability of Bioprosthetic Aortic Valves: Implications From 12,569 Implants. Ann. Thorac. Surg..

[33] Mack M.J., Leon M.B., Smith C.R., Mille C.D., Moses J.W., Tuzcu E.M., Webb J.G., Douglas P.S., Anderson W.N., Blackstone E.H. (2015). 5-year outcomes of transcatheter aortic valve replacement or surgical aortic valve re-placement for high surgical risk patients with aortic stenosis (PARTNER 1): A randomised controlled trial. Lancet.

[34] Barbanti M., Petronio A.S., Ettori F., Latib A., Bedogni F., De Marco F., Poli A., Boschetti C., De Carlo M., Fiorina C. (2015). 5-Year Outcomes After Transcatheter Aortic Valve Implantation with CoreValve Prosthesis. JACC Cardiovasc. Interv..

[35] Jørgensen T.H., Thyregod H.G.H., Ihlemann N., Nissen H., Petursson P., Kjeldsen B.J., Steinbrüchel D.A., Olsen P.S., Søndergaard L. (2021). Eight-year outcomes for patients with aortic valve stenosis at low surgical risk randomized to transcatheter vs. surgical aortic valve replacement. Eur. Heart J..

[36] Blackman D.J., Saraf S., MacCarthy P.A., Myat A., Anderson S.G., Malkin C.J., Cunnington M.S., Somers K., Brennan P., Manoharan G. (2019). Long-Term Durability of Transcatheter Aortic Valve Prostheses. J. Am. Coll. Cardiol..

[37] Généreux P., Piazza N., Alu M.C., Nazif T., Hahn R.T., Pibarot P., Bax J.J., Leipsic J.A., Blanke P., VARC-3 Writing Committee (2021). Valve Academic Research Consortium 3: Updated endpoint definitions for aortic valve clinical research. Eur. Heart J..

[38] Van Rosendael P.J., Delgado V., Bax J.J. (2018). Pacemaker implantation rate after transcatheter aortic valve implantation with early and new-generation devices: A systematic review. Eur. Heart J..

[39] Rodés-Cabau J., Ellenbogen K.A., Krahn A.D., Latib A., Mack M., Mittal S., Muntané-Carol G., Nazif T., Sondergaard L., Urena M. (2019). Management of Conduction Disturbances Associated with Transcatheter Aortic Valve Replacement. J. Am. Coll. Cardiol..

[40] Faroux L., Chen S., Muntané-Carol G., Regueiro A., Philippon F., Sondergaard L., Jørgensen T.H., Lopez-Aguilera J., Kodali S., Leon M. (2020). Clinical impact of conduction disturbances in transcatheter aortic valve replacement recipients: A systematic review and meta-analysis. Eur. Heart J..

[41] Pascual I., Hernández-Vaquero D., Alperi A., Almendarez M., Avanzas P., Kalavrouziotis D., Lorca R., Mesnier J., Arboine L., Mohammadi S. (2022). Permanent Pacemaker Reduction Using Cusp-Overlapping Projection in TAVR. JACC Cardiovasc. Interv..

[42] Faroux L., Guimaraes L., Wintzer-Wehekind J., Junquera L., Ferreira-Neto A.N., del Val D., Muntané-Carol G., Mohammadi S., Paradis J.-M., Rodés-Cabau J. (2019). Coronary Artery Disease and Transcatheter Aortic Valve Replacement. J. Am. Coll. Cardiol..

[43] Rotman O.M., Bianchi M., Ghosh R.P., Kovarovic B., Bluestein D. (2018). Principles of TAVR valve design, modelling, and testing. Expert Rev. Med. Devices.

[44] Yudi M.B., Sharma S.K., Tang G.H., Kini A. (2018). Coronary Angiography and Percutaneous Coronary Intervention after Transcatheter Aortic Valve Replacement. J. Am. Coll. Cardiol..

[45] Kim W.-K., Pellegrini C., Ludwig S., Möllmann H., Leuschner F., Makkar R., Leick J., Amat-Santos I.J., Dörr O., Breitbart P. (2021). Feasibility of Coronary Access in Patients with Acute Coronary Syndrome and Previous TAVR. JACC Cardiovasc. Interv..

[46] Faroux L., Lhermusier T., Vincent F., Nombela-Franco L., Tchétché D., Barbanti M., Abdel-Wahab M., Windecker S., Auffret V., Campanha-Borges D.C. (2021). ST-Segment Elevation Myocardial Infarction Following Transcatheter Aortic Valve Replacement. J. Am. Coll. Cardiol..

[47] Barbanti M., Costa G., Picci A., Criscione E., Reddavid C., Valvo R., Todaro D., Deste W., Condorelli A., Scalia M. (2020). Coronary Cannulation after Transcatheter Aortic Valve Replacement: The RE-ACCESS Study. JACC Cardiovasc. Interv..

[48] D’Ascenzo F., Verardi R., Visconti M., Conrotto F., Scacciatella P., Dziewierz A., Stefanini G.G., Paradis J.-M., Omedè P., Kodali S. (2018). Independent impact of extent of coronary artery disease and percutaneous revas-cularisation on 30-day and one-year mortality after TAVI: A meta-analysis of adjusted observational results. EuroIntervention.

[49] Sankaramangalam K., Banerjee K., Kandregula K., Mohananey D., Parashar A., Jones B.M., Jobanputra Y., Mick S., Krishnaswamy A., Svensson L.G. (2017). Impact of Coronary Artery Disease on 30-Day and 1-Year Mortality in Patients Undergoing Transcatheter Aortic Valve Replacement: A Meta-Analysis. J. Am. Heart Assoc..

[50] Van den Boogert T.P.W., Vendrik J., Claessen B.E.P.M., Baan J., Beijk M.A., Limpens J., Boekholdt S.A.M., Hoek R., Planken R.N., Henriques J.P. (2018). CTCA for detection of significant coronary artery disease in routine TAVI work-up: A systematic review and meta-analysis. Neth. Heart J..

[51] Mejía-Rentería H., Nombela-Franco L., Paradis J.-M., Lunardi M., Lee J.M., Amat-Santos I.J., Veiga Fernandez G., Kalra A., Bansal E.J., dela Tore Hernandez J.M. (2020). Angiography-based quantitative flow ratio versus fractional flow reserve in patients with coronary artery disease and severe aortic stenosis. EuroIntervention.

[52] Gohmann R.F., Pawelka K., Seitz P., Majunke N., Heiser L., Renatus K., Desch S., Lauten P., Holzhey D., Noack T. (2021). Combined cCTA and TAVR Planning for Ruling Out Significant CAD. JACC Cardiovasc. Imaging.

[53] Al-Lamee R., Thompson D., Dehbi H.-M., Sen S., Tang K., Davies J., Keeble T., Mielewczik M., Kaprielian R., Malik I.S. (2017). Percutaneous coronary intervention in stable angina (ORBITA): A double-blind, randomised controlled trial. Lancet.

[54] Maron D.J., Hochman J.S., Reynolds H.R., Bangalore S., O’Brien S.M., Boden W.E., Chaitman B.R., Senior R., López-Sendón J., Alexander K.P. (2020). Initial Invasive or Conservative Strategy for Stable Coronary Disease. N. Engl. J. Med..

[55] Chaitman B.R., Alexander K.P., Cyr D.D., Berger J.S., Reynolds H.R., Bangalore S., Boden W.E., Lopes R.D., Demkow W., Perna G.P. (2021). Myocardial Infarction in the ISCHEMIA Trial: Impact of Different Definitions on Incidence, Prognosis, and Treatment Comparisons. Circulation.

[56] Knuuti J., Wijns W., Saraste A., Capodanno D., Barbato E., Funck-Brentano C., Prescott E., Storey R.F., Deaton C., Cuisset T. (2020). 2019 ESC Guidelines for the diagnosis and management of chronic coronary syndromes. Eur. Heart J..

[57] Roberts W.C., Janning K.G., Ko J.M., Filardo G., Matter G.J. (2012). Frequency of Congenitally Bicuspid Aortic Valves in Patients ≥80 Years of Age Undergoing Aortic Valve Replacement for Aortic Stenosis (With or Without Aortic Regurgitation) and Implications for Transcatheter Aortic Valve Implantation. Am. J. Cardiol..

[58] Vincent F., Ternacle J., Denimal T., Shen M., Redfors B., Delhaye C., Simonato M., Debry N., Verdier B., Shahim B. (2021). Transcatheter Aortic Valve Replacement in Bicuspid Aortic Valve Stenosis. Circulation.

[59] Tchetche D., de Biase C., van Gils L., Parma R., Ochala A., Lefevre T., Hovasse T., De Backer O., Sondergaard L., Bleiziffer S. (2019). Bicuspid Aortic Valve Anatomy and Relationship with Devices: The BAVARD Mul-ticenter Registry: A European Picture of Contemporary Multidetector Computed Tomography Sizing for Bicuspid Valves. Circ. Cardiovasc. Interv..

[60] Yoon S.-H., Kim W.-K., Dhoble A., Pio S.M., Babaliaros V., Jilaihawi H., Pilgrim T., De Backer O., Bleiziffer S., Vincent F. (2020). Bicuspid Aortic Valve Morphology and Outcomes After Transcatheter Aortic Valve Replacement. J. Am. Coll. Cardiol..

[61] Abbasi M., Azadani A.N. (2015). Leaflet stress and strain distributions following incomplete transcatheter aortic valve expansion. J. Biomech..

[62] Tzemos N., Therrien J., Yip J., Thanassoulis G., Tremblay S., Jamorski M.T., Webb G.D., Siu S.C. (2008). Outcomes in Adults with Bicuspid Aortic Valves. JAMA.

[63] Wijesinghe N., Ye J., Rodés-Cabau J., Cheung A., Velianou J.L., Natarajan M.K., Dumont E., Nietlispach F., Gurvitch R., Wood D.A. (2010). Transcatheter Aortic Valve Implantation in Patients with Bicuspid Aortic Valve Stenosis. JACC Cardiovasc. Interv..

[64] Forrest J.K., Kaple R.K., Ramlawi B., Gleason T.G., Meduri C.U., Yakubov S.J., Jilaihawi H., Liu F., Reardon M.J. (2020). Transcatheter Aortic Valve Replacement in Bicuspid Versus Tricuspid Aortic Valves From the STS/ACC TVT Registry. JACC Cardiovasc. Interv..

[65] Makkar R.R., Yoon S.-H., Leon M.B., Chakravarty T., Rinaldi M., Shah P.B., Skipper E.R., Thourani V.H., Babaliaros V., Cheng W. (2019). Association Between Transcatheter Aortic Valve Replacement for Bicuspid vs Tricuspid Aortic Stenosis and Mortality or Stroke. JAMA.

[66] Fan J., Fang X., Liu C., Zhu G., Hou C.R., Jiang J., Lin X., Wang L., He Y., Zhu Q. (2020). Brain Injury After Transcatheter Replacement of Bicuspid Versus Tricuspid Aortic Valves. J. Am. Coll. Cardiol..

[67] Kalra A., Raza S., Hussain M., Shorbaji K., Delozier S., Deo S.V., Khera S., Kleiman N.S., Reardon M.J., Kolte D. (2019). Aortic Valve Replacement in Bioprosthetic Failure: Insights from The Society of Thoracic Surgeons National Database. Ann. Thorac. Surg..

[68] Webb J.G., Mack M.J., White J.M., Dvir D., Blanke P., Herrmann C.H., Leipsic J., Kodali S.K., Makkar R., Miller D.C. (2017). Transcatheter Aortic Valve Implantation Within Degenerated Aortic Surgical Biopros-theses: PARTNER 2 Valve-in-Valve Registry. J. Am. Coll. Cardiol..

[69] De Freitas Campos Guimarães L., Urena M., Wijeysundera H.C., Munoz-Garcia A., Serra C., Benitez L.M., Auffret V., Cheema A.N., Amat-Santos I.J., Fisher Q. (2018). Long-Term Outcomes after Transcatheter Aortic Valve-in-Valve Replacement. Circ. Cardiovasc. Interv..

[70] Tuzcu E.M., Kapadia S.R., Vemulapalli S., Carroll J.D., Holmes D.R., Mack M.J., Thourani V.H., Grover F.L., Brennan J.M., Suri R.M. (2018). Transcatheter Aortic Valve Replacement of Failed Surgically Implanted Bio-prostheses: The STS/ACC Registry. J. Am. Coll. Cardiol..

[71] Sá M.P.B.O., Van den Eynde J., Simonato M., Cavalcanti L.R.P., Doulamis I.P., Weixler V., Kampaktsis P.N., Gallo M., Laforgia P.L., Zhigalov K. (2021). Valve-in-Valve Transcatheter Aortic Valve Replacement Versus Redo Surgical Aortic Valve Replacement: An Updated Meta-Analysis. JACC Cardiovasc. Interv..

[72] Bleiziffer S., Simonato M., Webb J.G., Rodés-Cabau J., Pibarot P., Kornowski R., Windecker S., Erlebach M., Duncan A., Seiffert M. (2020). Long-term outcomes after transcatheter aortic valve implantation in failed biopros-thetic valves. Eur. Heart J..

[73] Ribeiro H.B., Rodés-Cabau J., Blanke P., Leipsic J., Park J.K., Bapat V., Makkar R., Simonato M., Barbanti M., Schofer J. (2017). Incidence, predictors, and clinical outcomes of coronary obstruction following transcatheter aortic valve replacement for degenerative bioprosthetic surgical valves: Insights from the VIVID registry. Eur. Heart J..

[74] Dvir D., Webb J.G., Bleiziffer S., Pasic M., Waksman R., Kodali S., Barbanti M., Latib A., Schaefer U., Rodés-Cabau J. (2014). Transcatheter Aortic Valve Implantation in Failed Bioprosthetic Surgical Valves. JAMA.

[75] Saxon J.T., Allen K.B., Cohen D.J., Chhatriwalla A.K. (2017). Bioprosthetic Valve Fracture during Valve-in-valve TAVR: Bench to Bedside. Interv. Cardiol. Rev. Res. Resour..

[76] Allen K.B., Chhatriwalla A.K., Saxon J.T., Sathananthan J., Dvir D., Webb J.G. (2020). Bioprosthetic valve fracture to facilitate valve-in-valve transcatheter aortic valve repair. Ann. Cardiothorac. Surg..

[77] Salem S.A., Foerst J.R. (2021). Valve-in-Valve Transcatheter Aortic Valve Replacement, with Present-Day Innovations and Up-to-Date Techniques. Interv. Cardiol. Clin..

[78] Khan J.M., Dvir D., Greenbaum A.B., Babaliaros V.C., Rogers T., Aldea G., Reisman M., Mackensen G.B., Eng M.H.K., Paone G. (2018). Transcatheter Laceration of Aortic Leaflets to Prevent Coronary Obstruction During Transcatheter Aortic Valve Replacement: Concept to First-in-Human. JACC Cardiovasc. Interv..

[79] Khan J.M., Greenbaum A.B., Babaliaros V.C., Rogers T., Eng M.H., Paone G., Leshnower B.G., Reisman M., Satler L., Waksman R. (2019). The BASILICA Trial: Prospective Multicenter Investigation of Intentional Leaflet Laceration to Prevent TAVR Coronary Obstruction. JACC Cardiovasc. Interv..

[80] De Backer O., Landes U., Fuchs A., Yoon S.-H., Mathiassen O.N., Sedaghat A., Kim W.-K., Pilgrim T., Buzzatti N., Ruile P. (2020). Coronary Access After TAVR-in-TAVR as Evaluated by Multidetector Computed Tomography. JACC Cardiovasc. Interv..

[81] Landes U., Webb J.G., De Backer O., Sondergaard L., Abdel-Wahab M., Crusius L., Kim W.-K., Hamm C., Buzzatti N., Montorfano M. (2020). Repeat Transcatheter Aortic Valve Replacement for Transcatheter Prosthesis Dys-function. J. Am. Coll. Cardiol..

[82] Greenbaum A.B., Kamioka N., Vavalle J.P., Lisko J.C., Gleason P.T., Paone G., Grubb K.J., Bruce C.G., Lederman R.J., Babaliaros V.C. (2021). Balloon-Assisted BASILICA to Facilitate Redo TAVR. JACC Cardiovasc. Interv..

[83] Dallan L.A.P., Forrest J.K., Reardon M.J., Szeto W.Y., George I., Kodali S., Kleiman N.S., Yakubov S.J., Grubb K.J., Liu F. (2021). Transcatheter Aortic Valve Replacement with Self-Expandable Supra-Annular Valves for Degenerated Surgical Bioprostheses: Insights from Transcatheter Valve Therapy Registry. J. Am. Heart Assoc..

[84] Auffret V., Lefevre T., VAN Belle E., Eltchaninoff H., Iung B., Koning R., Motreff P., Leprince P., Verhoye J.P., Manigold T. (2017). Temporal Trends in Transcatheter Aortic Valve Replacement in France. J. Am. Coll. Cardiol..

[85] Grover F.L., Vemulapalli S., Carroll J.D., Edwards F.H., Mack M.J., Thourani V.H., Brindis R.G., Shahian D.M., Ruiz C.E., Jacobs J.P. (2016). 2016 Annual Report of The Society of Thoracic Surgeons/American College of Cardiology Transcatheter Valve Therapy Registry. J. Am. Coll. Cardiol..

[86] Dahle T.G., Kaneko T., McCabe J.M. (2019). Outcomes Following Subclavian and Axillary Artery Access for Transcatheter Aortic Valve Replacement. JACC Cardiovasc. Interv..

[87] Chamandi C., Akar R.A., Rodés-Cabau J., Blanchard D., Dumont E., Spaulding C., Doyle D., Pagny J.-Y., DeLarochellière R., Lafont A. (2018). Transcarotid Compared with Other Alternative Access Routes for Transcatheter Aortic Valve Replacement. Circ. Cardiovasc. Interv..

[88] Blackstone E.H., Suri R.M., Rajeswaran J., Babaliaros V., Douglas P.S., Fearon W.F., Miller D.C., Hahn R.T., Kapadia S., Kirtane A.J. (2015). Propensity-Matched Comparisons of Clinical Outcomes After Transapical or Transfemoral Transcatheter Aortic Valve Replacement: A Placement of Aortic Transcatheter Valves (PARTNER)-I Trial Substudy. Circulation.

[89] Beurtheret S., Karam N., Resseguier N., Houel R., Modine T., Folliguet T., Chamandi C., Com O., Gelisse R., Bille J. (2019). Femoral Versus Nonfemoral Peripheral Access for Transcatheter Aortic Valve Replacement. J. Am. Coll. Cardiol..

[90] Overtchouk P., Folliguet T., Pinaud F., Fouquet O., Pernot M., Bonnet G., Hubert M., Lapeze J., Claudel J.P., Ghostine S. (2019). Transcarotid Approach for Transcatheter Aortic Valve Replacement with the Sapien 3 Prosthesis. JACC Cardiovasc. Interv..

[91] Gleason T.G., Schindler J.T., Hagberg R.C., Deeb M., Adams D.H., Conte J.V., Zorn G.L., Hughes G.C., Guo J., Popma J.J. (2018). Subclavian/Axillary Access for Self-Expanding Transcatheter Aortic Valve Re-placement Renders Equivalent Outcomes as Transfemoral. Ann. Thorac. Surg..

[92] Iung B., Baron G., Butchart E.G., Delahaye F., Gohlke-Bärwolf C., Levang O.W., Tornos P., Vanoverschelde J.-L., Vermeer F., Boersma E. (2003). A prospective survey of patients with valvular heart disease in Europe: The Euro Heart Survey on Valvular Heart Disease. Eur. Heart J..

[93] Franzone A., Piccolo R., Siontis G.C., Lanz J., Stortecky S., Praz F., Roost E., Vollenbroich R., Windecker S., Pilgrim T. (2016). Transcatheter Aortic Valve Replacement for the Treatment of Pure Native Aortic Valve Regurgitation. JACC Cardiovasc. Interv..

[94] Poschner T., Werner P., Kocher A., Laufer G., Musumeci F., Andreas M., Russo M. (2022). The JenaValve pericardial transcatheter aortic valve replacement system to treat aortic valve disease. Future Cardiol..

[95] Gogia S., Vahl T., Khalique O., Hamid N., Borden S., Chung C., Ng V., Patel A., George I., Hahn R. (2020). TCT CONNECT-92 Initial Single-Center Experience with Transfemoral Transcatheter Aortic Valve Replacement in Patients with Symptomatic Severe Aortic Regurgitation. J. Am. Coll. Cardiol..

[96] Hensey M., Murdoch D.J., Sathananthan J., Alenezi A., Sathananthan G., Moss R., Blanke P., Leipsic J., Wood D.A., Cheung A. (2019). First-in-human experience of a new-generation transfemoral transcatheter aortic valve for the treatment of severe aortic regurgitation: The J-Valve transfemoral system. EuroIntervention.

[97] Barbanti M., Ye J., Pasupati S., El-Gamel A., Webb J.G. (2013). The Helio transcatheter aortic dock for patients with aortic regurgitation. EuroIntervention.

